# Cyclin D1 extensively reprograms metabolism to support biosynthetic pathways in hepatocytes

**DOI:** 10.1016/j.jbc.2023.105407

**Published:** 2023-10-28

**Authors:** Heng Wu, Betsy T. Kren, Andrew N. Lane, Teresa A. Cassel, Richard M. Higashi, Teresa W.M. Fan, George S. Scaria, Laurie L. Shekels, Mark A. Klein, Jeffrey H. Albrecht

**Affiliations:** 1 Division of Gastroenterology, Hepatology, and Nutrition, University of Minnesota, Minneapolis, Minnesota, USA; 2 Research Service, Minneapolis VA Health Care System, Minneapolis, Minnesota, USA; 3 Center for Environmental and Systems Biochemistry, Department of Toxicology and Cancer Biology, and Markey Cancer Center, University of Kentucky, Lexington, Kentucky, USA; 4 Hematology and Oncology Division, Minneapolis VA Health Care System, Minneapolis, Minnesota, USA

**Keywords:** aldolase, anaerobic glycolysis, BAY 2402234, cell cycle, cyclin D1, glyceraldehyde-3-phosphate dehydrogenase (GAPDH), liver regeneration, palbociclib, pentose phosphate pathway (PPP), purine, pyrimidine

## Abstract

Cell proliferation requires metabolic reprogramming to accommodate biosynthesis of new cell components, and similar alterations occur in cancer cells. However, the mechanisms linking the cell cycle machinery to metabolism are not well defined. Cyclin D1, along with its main partner cyclin-dependent kinase 4 (Cdk4), is a pivotal cell cycle regulator and driver oncogene that is overexpressed in many cancers. Here, we examine hepatocyte proliferation to define novel effects of cyclin D1 on biosynthetic metabolism. Metabolomic studies reveal that cyclin D1 broadly promotes biosynthetic pathways including glycolysis, the pentose phosphate pathway, and the purine and pyrimidine nucleotide synthesis in hepatocytes. Proteomic analyses demonstrate that overexpressed cyclin D1 binds to numerous metabolic enzymes including those involved in glycolysis and pyrimidine synthesis. In the glycolysis pathway, cyclin D1 activates aldolase and GAPDH, and these proteins are phosphorylated by cyclin D1/Cdk4 *in vitro*. *De novo* pyrimidine synthesis is particularly dependent on cyclin D1. Cyclin D1/Cdk4 phosphorylates the initial enzyme of this pathway, carbamoyl-phosphate synthetase 2, aspartate transcarbamylase, and dihydroorotase (CAD), and metabolomic analysis indicates that cyclin D1 depletion markedly reduces the activity of this enzyme. Pharmacologic inhibition of Cdk4 along with the downstream pyrimidine synthesis enzyme dihydroorotate dehydrogenase synergistically inhibits proliferation and survival of hepatocellular carcinoma cells. These studies demonstrate that cyclin D1 promotes a broad network of biosynthetic pathways in hepatocytes, and this model may provide insights into potential metabolic vulnerabilities in cancer cells.

Compared to the quiescent state, cells undergoing proliferation and growth manifest profound metabolic alterations to generate amino acids, nucleotides, and other precursors necessary to synthesize macromolecules required for increased cell mass (1, 2). Similar metabolic reprogramming is a hallmark of malignant cells, which demonstrate enhanced nutrient uptake, increased biosynthesis, adaptations to redox stress, and other biochemical adaptations (3, 4, 5). However, the mechanisms by which cell cycle progression, growth, and anabolic metabolism are coordinated remain incompletely understood (6).

Glucose metabolism is markedly altered during proliferation, and this is a key feature of cancer cell metabolism (1, 2, 3, 4, 5). Proliferating cells generally have increased utilization of glucose *via* glycolysis, but much of this does not undergo full oxidation in the mitochondria. Rather, glycolytic intermediates are shunted into biosynthetic pathways that produce substrates for cell growth. Pyruvate, the end product of glycolysis, is reduced to lactate which is excreted from the cell. This metabolic feature of proliferating cells, called the Warburg effect, appears to play an important role in cancer cell metabolism (3, 4, 5). A number of oncogene and tumor suppressor proteins have been shown to modulate glycolysis and other aspects of metabolism, but there is little information about how these pathways are regulated by cell cycle proteins.

Hepatocytes present a unique system to study the interaction between cell cycle progression, tissue growth, and metabolism. The liver performs a wide range of essential metabolic functions including glucose homeostasis (7). Hepatocytes rarely proliferate under normal circumstances, but can readily enter the cell cycle in response to injuries that diminish functional liver mass. For example, after two-thirds partial hepatectomy (PH) in rodents, differentiated hepatocytes rapidly proliferate and liver mass is restored within 1 to 2 weeks; this is one of the most striking examples of physiologically regulated cell proliferation (8, 9). In patients with acute and chronic liver diseases, compensatory hepatocyte proliferation is a determinant of survival (10, 11). Although liver regeneration has been extensively studied, the mechanisms that regulate the balance between homeostatic and biosynthetic metabolism are not well characterized.

The cell cycle is regulated by protein kinase complexes consisting of cyclins and cyclin-dependent kinases (Cdks). In many cell types including hepatocytes, cyclin D1 is expressed at low levels in quiescent cells and markedly upregulated in G1 phase by mitogenic stimuli, whereupon it binds and activates its main partner, Cdk4. Depletion or KO of cyclin D1 inhibits hepatocyte proliferation (12, 13, 14). Interestingly, transient expression of cyclin D1 is sufficient to induce robust hepatocyte proliferation and marked liver growth, even under conditions that normally inhibit these processes (15, 16, 17). These findings indicate that cyclin D1 can rapidly reprogram hepatocyte metabolism to promote biosynthesis required for cell growth.

Prior studies have shown that cyclin D1 inhibits many aspects of homeostatic metabolism in hepatocytes, in part by downregulating the activity of key metabolic transcriptional regulators such as hepatocyte nuclear factor 4α (HNF4α), peroxisome proliferator-activated receptor α (PPARα), carbohydrate response element binding protein (ChREBP, gene name Mlxipl), and peroxisome proliferator-activated receptor-γ coactivator α (PGC-1α) (12, 14, 18, 19, 20), thus potentially allowing cellular resources to be redirected to the demands of growth and proliferation. However, there is little information about how this protein may promote anabolic metabolism in hepatocytes or other cells. Importantly, in addition to its role in normal cell cycle progression, cyclin D1 is one of the most commonly overexpressed genes in human malignancies including hepatocellular carcinoma (HCC) (21, 22), and thus understanding its metabolic effects may provide insight into novel approaches to cancer therapy.

To investigate how cyclin D1 regulates biosynthetic metabolism in hepatocytes, we used metabolomics and proteomics approaches to identify specific pathways modulated by this protein. By tracking the fate of ^13^C-glucose, stable isotope-resolved metabolomics revealed that cyclin D1 depletion in hepatocytes markedly inhibited biosynthetic pathways including glycolysis, the pentose phosphate pathway (PPP), and purine and pyrimidine synthesis. Proteomic analysis of cyclin D1-binding proteins from the liver showed that numerous metabolic enzymes bind to this cell cycle protein, including those involved in key biosynthetic pathways. These findings indicate that cyclin D1 promotes several anabolic pathways, which provides new insight into the mechanisms by which this key cell cycle protein and oncogene regulates proliferation and growth.

## Results

### Cyclin D1 regulates glycolysis and mitochondrial respiration

**Figure 1:**
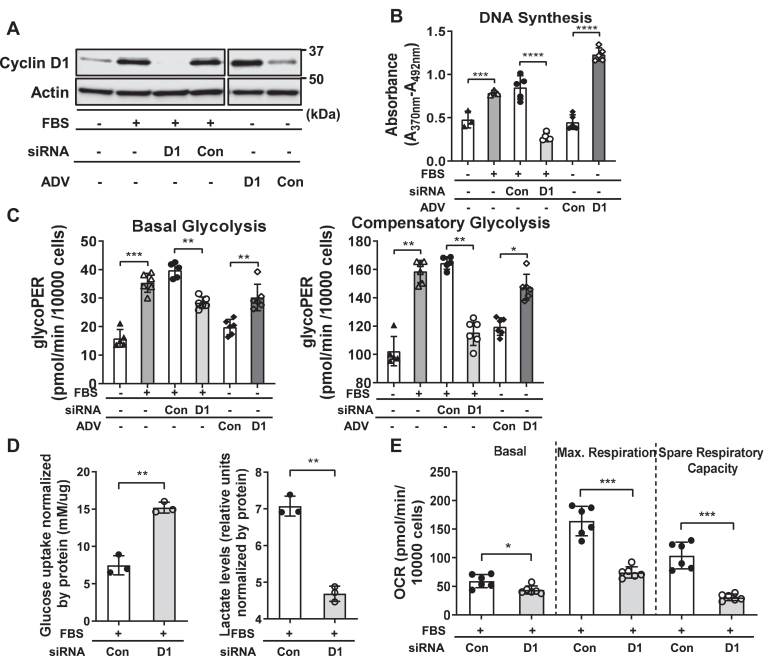
**Modulation of glycolysis and mitochondrial respiration in live hepatocytes.** Mouse AML12 hepatocytes were cultured in the presence or absence of 10% fetal bovine serum (FBS). Cells were treated with cyclin D1 (or control) siRNA or transduced with an adenovirus overexpressing cyclin D1 (or control) as indicated, and harvested after 48 h. *A*, Western blot of cyclin D1 expression. *B*, DNA synthesis are measured by BrdU uptake. *C*, basal and compensatory glycolysis as measured by ECAR. *D*, glucose uptake and media lactate content (normalized to cellular protein content). *E*, basal and maximal respiration and spare respiratory capacity as measured by OCR (∗*p* < 0.05; ∗∗*p* < 0.01; and ∗∗∗*p* < 0.001). ECAR, extracellular acidification rate; OCR, oxygen consumption rate.

We have recently demonstrated that cyclin D1 depletion or KO leads to increased hepatocyte uptake of glucose and its incorporation into glycogen, indicating that this protein diverts glucose away from energy storage (14). To initially examine how cyclin D1 regulates glycolysis and cellular energetics, we used mouse AML12 hepatocytes stimulated with mitogen (10% serum) along with siRNA-mediated depletion or adenovirus (ADV)-mediated overexpression of this protein. As previously shown (12, 13, 14), serum stimulates cyclin D1 expression and cell cycle progression (as measured by BrdU incorporation to assess DNA synthesis), whereas siRNA-mediated knockdown of this protein inhibits these (Fig. 1, *A* and *B*). In the absence of serum, transduction with an ADV expressing cyclin D1 (ADV-D1) promotes hepatocyte cell cycle progression. As a measure of glycolysis, the extracellular acidification rate (ECAR) was monitored using an Agilent Seahorse analyzer. Mitogenic stimulation with serum increased basal and compensatory glycolysis, and these were significantly inhibited by cyclin D1 depletion (Fig. 1*C*). Conversely, in serum-deprived cells, cyclin D1 transduction stimulated glycolysis. As a direct measure of glycolysis, media lactate levels were measured, which were decreased with cyclin D1 siRNA (Fig. 1*D*). Thus, in hepatocytes, cyclin D1 appears to be necessary for mitogen-stimulated glycolysis and is sufficient to promote this in the absence of mitogens.

We also examined whether cyclin D1 similarly regulated glycolysis in cancer cell lines by measuring lactate production. In serum-stimulated liver cancer (HepG2, HuH7, and SK-Hep1) and breast cancer (MCF7) cells, knockdown of cyclin D1 led to decreased cell cycle progression (as measured by DNA synthesis) and diminished lactate production (Fig. S1). Interestingly, cyclin D1 had variable effects on glucose uptake. In cancer cells derived from hepatocytes (HepG2 and HuH7) and breast epithelia (MCF 7), cyclin D1 depletion increased glucose uptake. We speculate that this protein may repress glucose uptake and its incorporation into energy storage (*i.e.*, glycogen synthesis and *de novo* synthesis of triglyceride) in cancer cells derived from organs that normally perform these functions, as we have previously shown in hepatocytes (14, 18). In the Sk-Hep1 cell line that likely arose from liver sinusoidal endothelial cells (23), cyclin D1 knockdown reduced glucose uptake. Regardless of its effect on glucose uptake, in each cell line examined, cyclin D1 siRNA significantly inhibited lactate production, further indicating that this protein stimulates the Warburg effect.

To assess mitochondrial respiration, we measured the oxygen consumption rate (OCR) in hepatocytes (Fig. 1*E*). Basal OCR was moderately decreased by cyclin D1 siRNA, whereas spare respiratory capacity was markedly inhibited. Spare respiratory capacity is a measure of mitochondrial reserve and enhances cellular resilience in the setting of metabolic and oxidative stress and is largely dependent on the oxidation of either pyruvate derived from glycolysis or fatty acids (24). Our prior studies have shown that cyclin D1 depletion enhances lipolysis and fatty acid oxidation (12, 13), suggesting that the decrease in OCR observed with its knockdown under the conditions used here (with no supplemental lipids in the media) reflects decreased availability of pyruvate from glycolysis or decreased utilization of glutamine for mitochondrial oxidative metabolism (25).

### Cyclin D1 binds to metabolic enzymes involved in diverse pathways

Our prior studies found that cyclin D1 regulates the expression of a broad range of metabolic enzymes at the mRNA and/or protein expression level in liver, hepatocytes, and liver cancer cell lines (14, 26). We surmised that it may also regulate key metabolic pathways through direct protein-protein interaction. To investigate this, we transduced the mouse liver with an ADV encoding human cyclin D1 with hemagglutinin (HA) and FLAG epitope tags (ADV-D1-HA-FLAG) for 1 day, which promotes robust hepatocyte proliferation (15, 16, 17). Liver lysates were used for sequential purification of FLAG- and HA-binding proteins, followed by proteomic analysis by mass spectrometry (MS) to identify binding proteins. A liver transduced with ADV-cyclin D1 (with no tags) was processed identically to serve as a negative control for nonspecific binding; in this sample only keratins were recovered, which are considered to be contaminants. Approximately, 140 proteins were identified following tandem affinity purification of cyclin D1-HA-FLAG (Table S1).

**Figure 2:**
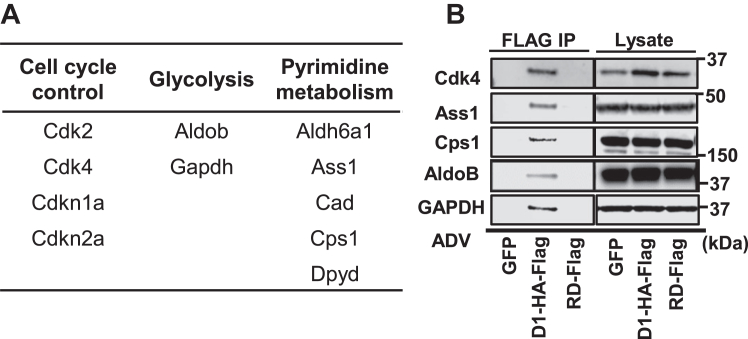
**Cyclin D1 binds metabolic proteins in the liver.** Livers were transduced with cyclin D1-HA-FLAG (or a control vector encoding GFP), and lysates were subjected to tandem affinity purification followed by mass spectrometry. *A*, selected cell cycle proteins and metabolic enzymes binding to cyclin D1 by mass spectroscopy. The mouse gene name is shown for each. *B*, lysates of livers transduced with either full-length cyclin D1-HA-FLAG or a truncation mutant of the midportion of cyclin D1 (RD-FLAG (14)) were subjected to immunoprecipitation and elution from anti-FLAG beads as described in the Experimental procedures. The eluted proteins and the corresponding lysates were subjected to Western blot analysis of the indicated proteins. HA, hemagglutinin.

As expected, cyclin D1 bound to cell cycle proteins in the liver such as Cdk4, p21 (Cdkn1a), and p27 (Cdkn1b), as we have previously shown by immunoprecipitation-Western blot (27, 28) (Fig. 2*A*). Cyclin D1 bound to groups of proteins involved in numerous other cellular processes including transcription, RNA processing, translation, protein degradation, and intracellular transport. Interestingly, more than 45 of the proteins bound to cyclin D1 were metabolic enzymes involved in diverse pathways. For example, enzymes involved in glycolysis and pyrimidine metabolism were bound to cyclin D1 by the proteomic analysis (Fig. 2*A*). The proteins shown in Figure 2*B* bound to full-length cyclin D1 but not a truncation mutant containing only the midportion of cyclin D1 (called the RD mutant), which binds to several nuclear receptor transcription factors but not Cdk4 (18, 29). This suggests that other domains of cyclin D1 mediate the binding to these enzymes.

### Cyclin D1 stimulates aldolase and GAPDH activity

**Figure 3:**
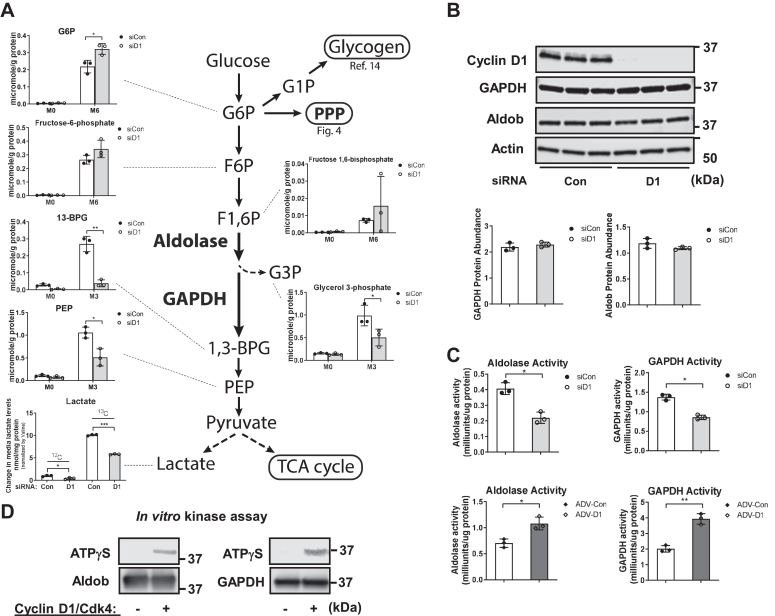
**Analysis of the glycolytic pathway.** AML12 hepatocytes were cultured as in Figure 1 in the presence of serum and [^13^C_6_]-glucose for 24 h, followed by MS analysis of cell extracts and NMR of media (n = 3 per condition). *A*, abundance of key ^13^C isotopologues of glycolytic metabolites by IC-MS and media lactate by NMR. Values are normalized to cellular protein content for each replicate. *B*, Western blot of aldolase B and GAPDH. *C*, aldolase and GAPDH activity assays in cells with cyclin D1 siRNA (*top*), or in serum-deprived cells transduced with cyclin D1 as in Figure 1 (ADV-D1, *bottom*). *D*, nonradioactive *in vitro* kinase assay using recombinant cyclin D1/Cdk4, aldolase B, and GAPDH along with ATPγS. Western blot was performed using an antibody to thiophosphate ester (*top*, representing the phosphorylated protein) or the substrate proteins (*bottom*). ADV-D1, adenovirus expressing cyclin D1; Cdk4, cyclin-dependent kinase 4; IC-MS, ion chromatography with mass spectrometry; MS, mass spectrometry.

To gain a broader understanding of how cyclin D1 regulates cellular metabolism, we performed unbiased metabolic profiling of AML12 hepatocytes labeled with ^13^C-glucose (Fig. 3). Stable isotope-resolved metabolomics can provide unique insight into the regulation of specific steps in multiple metabolic pathways (30, 31, 32). Hepatocytes were incubated with [^13^C_6_]-glucose for 1 day prior to harvest, and extracts were prepared for analysis by MS and NMR.

Figure 3*A* shows the abundance of key metabolites in the glycolytic pathway. We have recently shown that cyclin D1 knockdown or KO in hepatocytes markedly promotes glucose uptake and its incorporation into glycogen (14). The abundance of glucose-6-phosphate (G6P) was modestly increased by cyclin D1 siRNA (14), and there was no significant change in the downstream glycolytic metabolites fructose-6-phosphate and fructose-1,6-biphosphosphate (F1,6P). However, there was substantially decreased ^13^C_3_-labeling of 3-carbon products downstream in the glycolytic pathway including 1,3-bisphosphoglycerate, phosphoenolpyruvate, and glycerol 3-phosphate. Furthermore, cyclin D1 depletion inhibited ^13^C_3_-labeled lactate production (in agreement with the lactate assay in Fig. 1*E*).

The metabolomic analysis above suggest that cyclin D1 is required for optimal activation of the aldolase and/or GAPDH steps of glycolysis. Western blot analysis of aldolase B (Aldob, the primary aldolase form in hepatocytes) and GAPDH did not show a corresponding decrease in the expression of these proteins (Fig. 3*B*). Notably, the unbiased proteomic analysis in Figure 2 showed that cyclin D1 binds to Aldob and GAPDH in the liver, suggesting that it may regulate these enzymes through protein-protein binding or phosphorylation. Similarly, prior proteomic studies found that cyclin D1 binds to aldolase and GAPDH, but did not pursue these findings (33, 34, 35). To examine this further, we performed assays of aldolase and GAPDH activities using standard commercial kits, and these were significantly decreased by cyclin D1 knockdown (Fig. 3*C*, top). Conversely, in serum-deprived cells, ectopic expression of cyclin D1 (using ADV-D1 as in Fig. 1) promoted the activity of these enzymes (Fig. 3*C*, bottom). To evaluate the possibility that cyclin D1/Cdk4 regulates these enzymes by phosphorylation, we performed nonradioactive kinase assays using recombinant proteins (Fig. 3*D*). These showed phosphorylation of both Aldob and GAPDH by cyclin D1/Cdk4, suggesting a potential posttranslational mechanism by which cyclin D1 activates these key steps of glycolysis.

Another potential regulatory step of glycolysis is at the level of phosphofructokinase-1 (PFK1), which catalyzes the conversion of fructose-6-phosphate + ATP to F1,6P + ADP. PFK1 activity is controlled through complex mechanisms including allosteric inhibition by ATP, phosphoenolpyruvate, and citrate and activation by ADP, AMP, and fructose 2,6-bisphosphate. Interestingly, in proliferating human leukemia cells, the closely related cyclin D3/Cdk6 complex phosphorylates and inhibits PFK1 (36). The abundance of F1,6P varied substantially between samples in our analysis (Fig. 3*A*), but did not clearly decrease, suggesting that inhibition of glycolysis by cyclin D1 knockdown was not primarily due to inhibition of PFK1. The expression of PFK isoforms (Pfkl, Pfkm, and Pkfp) were unchanged or modestly reduced by cyclin D1 siRNA (Fig. S2*A*). Further, the ATP/ADP and ATP/AMP ratios, which regulate PFK1 activity, were not significantly altered (Fig. S2*B*). Thus, while we cannot rule out the possibility that cyclin D1 affects PFK1 under these conditions, the data support the concept that it regulates glycolysis *via* activation of aldolase and GAPDH.

### Cyclin D1 promotes PPP activity

**Figure 4:**
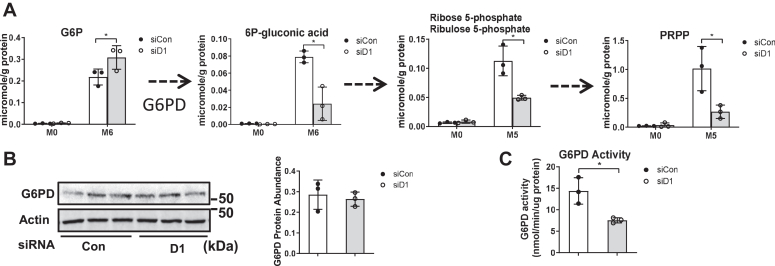
**Regulation of the PPP by cyclin D1.** Hepatocytes were cultured as in Figure 3. *A*, the abundance of unlabeled and fully ^13^C labeled (M6 or M5) PPP metabolites as determined by IC-UHR FTMS. *B*, Western blot of G6PD. *C*, G6PD enzyme activity assay using a commercial kit. G6PD, glucose-6-phosphate dehydrogenase; IC-UHR FTMS, ion chromatography coupled with ultra high-resolution Fourier transform mass spectrometry; PPP, pentose phosphate pathway.

Another key metabolic feature of proliferating and malignant cells is the activation of the PPP, which provides the pentose phosphoribosyl pyrophosphate (PRPP) for nucleic acid synthesis and NADPH for maintaining cellular redox balance (37, 38). The initial substrate in the oxidative branch of this pathway, G6P, was modestly increased by cyclin D1 knockdown (14). In contrast, downstream of the initial rate-limiting initial enzyme of this pathway, glucose-6-phosphate dehydrogenase (G6PD), each metabolite was decreased by cyclin D1 siRNA (Fig. 4*A*). The expression of G6PD did not change with cyclin D1 depletion (Fig. 4*B*), but its enzyme activity was decreased (Fig. 4*C*), which corroborates the metabolomics data. These findings suggest that cyclin D1 promotes G6PD activity *via* posttranslational modification(s) or allosteric mechanisms (*e.g.*, by altered NADPH levels), which are known to play a key role in regulating the function of this enzyme (37, 38).

### Cyclin D1 activates the *de novo* purine synthesis pathway

**Figure 5:**
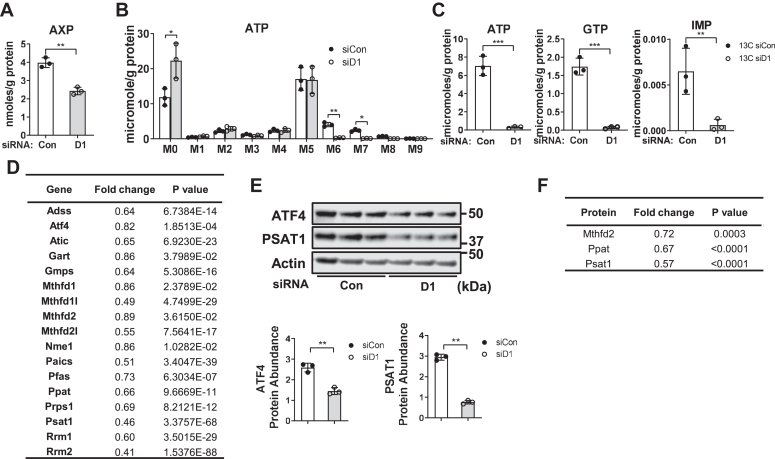
**Cyclin D1 promotes purine synthesis.** Hepatocytes were cultured as in Figure 3. *A*, abundance of ^13^C labeled adenine in AXP (AMP, ADP, and ATP combined) by NMR. *B*, ^13^C-labeled isotopologues of ATP by IC-UHR FTMS. *C*, abundance of ^13^C-enriched purine rings of ATP, GFP, and IMP, calculated by the sum of the ^13^C M6–M8 isotopologues. *D*, changes in the level of mRNA transcripts of genes involved in purine synthesis induced by cyclin D1 siRNA (relative to control siRNA) from our prior RNA-seq study in AML12 hepatocytes cultured under these conditions (ref. (14)). *E*, Western blot of ATF4 and Psat1. Relative expression is shown in the graphs below. *F*, expression of Mthfd2, Ppat, and Psat1 proteins by reverse phase protein array analysis. IC-UHR FTMS, ion chromatography coupled with ultra high-resolution Fourier transform mass spectrometry.

Cell proliferation requires increased nucleotide synthesis for the production of DNA, RNA, and other key metabolites. Purine nucleotide synthesis results from the assembly of a purine ring directly on the one position of PRPP, using metabolites arising from the serine/glycine and one-carbon pathways (39, 40). As detected by NMR, the abundance of [U-^13^C] glucose-derived adenine in AXP (AMP, ADP, and ATP combined) was significantly reduced by cyclin D1 depletion (Fig. 5*A*). Ion chromatography coupled with ultra high-resolution Fourier transform mass spectrometry (IC-UHR FTMS) analysis demonstrated nearly complete inhibition of ^13^C_6–8_ (M6–M8) but not ^13^C_5_ (M5) isotopologues of ATP, which represent the adenine and ribose rings, respectively (Fig. 5, *B* and *C*). Similarly, the abundance of ^13^C-labeled purine rings of GTP and the common purine precursor IMP were greatly diminished (Fig. 5*C*). These data indicate marked inhibition of the purine ring synthesis pathway but not ribose incorporation into purine nucleotides.

Using data from our prior RNA-seq analysis of AML12 cells treated with cyclin D1 siRNA under these conditions (14), we examined the expression of genes involved in this pathway (40, 41). Cyclin D1 knockdown broadly reduced expression of transcripts involved in the serine/glycine pathway, one-carbon metabolism, and purine ring synthesis (Fig. 5*D*). Similarly, expression of key proteins involved in purine synthesis (ATF4, Psat1, Ppat, and Mthfd2 (41)) were reduced in the absence of cyclin D1 (Fig. 5, *E* and *F*). These results suggest that cyclin D1 promotes purine ring synthesis by upregulating the expression of enzymes involved in multiple steps in the process.

### Cyclin D1 promotes pyrimidine synthesis and CAD activity

**Figure 6:**
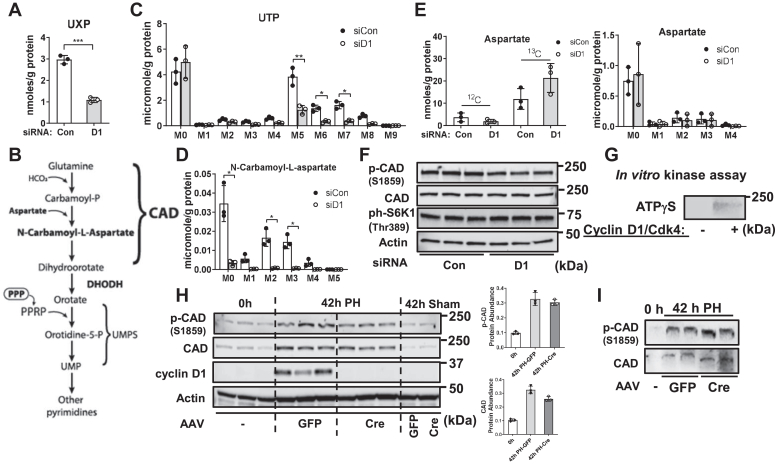
**Regulation of pyrimidine synthesis and CAD by cyclin D1.** Hepatocytes were cultured as in Figure 3. *A*, amount of ^13^C-UXP (UMP, UDP, and UTP combined) at 5 and 6 positions by NMR. *B*, diagram of the pyrimidine synthesis pathway. *C*, abundance of ^13^C isotopologues of UTP by MS. *D*, concentration of N- carbamoyl-L-aspartate isotopologues by MS. *E*, abundance of ^13^C-aspartate by NMR (*left*) and MS (*right*). *F*, expression of total CAD, phospho-Ser1859 CAD, and ph-S6K1 by Western blot in AML12 hepatocytes. *G*, nonradioactive *in vitro* kinase assay using recombinant cyclin D1/Cdk4 along with CAD-HA-FLAG isolated from fasting mouse liver and ATPγS. Western blot was performed using an antibody to thiophosphate ester. *H*, Western blot analysis of resting (0 h) and regenerating liver 42 h after PH in control (GFP) or hepatocyte-specific KO (Cre) mice. The relative expression of total and phospho-Ser1859 CAD are shown. *I*, Phos-Tag Western blot of total and phospho-Ser1859 CAD from the PH model. Cyclin D1 KO (Cre) was associated with a less phosphorylated form of total and phospho-Ser1859 CAD compared to control mice (GFP). CAD, carbamoyl-phosphate synthetase 2, aspartate transcarbamylase, and dihydroorotase; Cdk4, cyclin-dependent kinase 4; HA, hemagglutinin; MS, mass spectrometry; PH, partial hepatectomy.

One of the most striking findings in the metabolomics data was that cyclin D1 depletion markedly inhibited pyrimidine nucleotide synthesis. In hepatocytes incubated with [U-^13^C]-glucose, NMR revealed a significant decrease in the ^13^C labeling of pyrimidines UXP (UMP, UDP, and UTP combined) at the 5 and 6 positions of the uracil ring derived from [U-^13^C]-glucose (Fig. 6*A*). As shown in Figure 6*C*, IC-UHR FTMS revealed that all major ^13^C-labeled isotopologues in UTP derived from glucose were significantly decreased by cyclin D1 siRNA (similar findings were observed for other pyrimidines, data not shown). The decrease in M5-labeled UTP represents decreased incorporation of PRPP derived from the PPP. The substantially decreased M6–M8 isotopologues represent diminished pyrimidine ring synthesis.

The decrease in pyrimidine ring production with cyclin D1 siRNA was associated with the nearly complete disappearance of the key intermediate metabolite N-carbamoyl-L-aspartate (Fig. 6*D*), a product of carbamoyl-phosphate synthetase 2, aspartate transcarbamylase, and dihydroorotase (CAD), a multifunctional enzyme that catalyzes the first committed steps of *de novo* pyrimidine synthesis (Fig. 6*B*) (42). Independently, our proteomics study also indicate that cyclin D1 binds to CAD (Fig. 2*A*), which was also noted (but not pursued) in a separate proteomics analysis (34). Notably, cyclin D1 siRNA did not decrease the abundance of ^13^C-labeled aspartate (Fig. 6*E*), suggesting that substrate availability was not limiting. These data suggest that cyclin D1 promotes the activity of CAD.

Prior studies have shown that CAD is activated by phosphorylation at Ser1859 by S6K1 downstream of mechanistic target of rapamycin complex 1 (TORC1) (43, 44). In AML12 cells, cyclin D1 knockdown did not significantly affect the abundance of either total or ph-Ser1859 CAD (Fig. 6*F*). This led us to investigate whether cyclin D1 might promote CAD activity by phosphorylation at a distinct site. Recombinant cyclin D1/Cdk4 phosphorylated CAD in an *in vitro* kinase reaction (Fig. 6*G*). We also examined the regulation of CAD following two-thirds PH (a model of robust hepatocyte proliferation (8, 9)), in mice with acute hepatocyte-specific KO of cyclin D1 (these animals and experiments are described in ref. (14)). Expression of both CAD and ph-Ser1859 CAD were markedly upregulated in regenerating mouse liver after PH in both control and hepatocyte-specific cyclin D1 KO mice by standard Western blot (Fig. 6*H*). However, using a Phos-Tag Western blot, which accentuates phosphorylation changes (45), we noted that hepatocyte-specific cyclin D1 KO mice had a less-phosphorylated form of both total and ph-Ser1859 CAD (Fig. 6*I*–phosphorylated forms migrate more slowly using this technique). These data suggest that cyclin D1 regulates phosphorylation of CAD at a site distinct from Ser1859. Although further studies are required to identify the potential cyclin D1/Cdk4 phosphorylation site(s) of CAD, and their functional significance, these data support the concept that cyclin D1 promotes pyrimidine synthesis by activating CAD *via* phosphorylation.

### Combined inhibition of Cdk4 and DHODH leads to HCC cell growth inhibition and death

**Figure 7:**
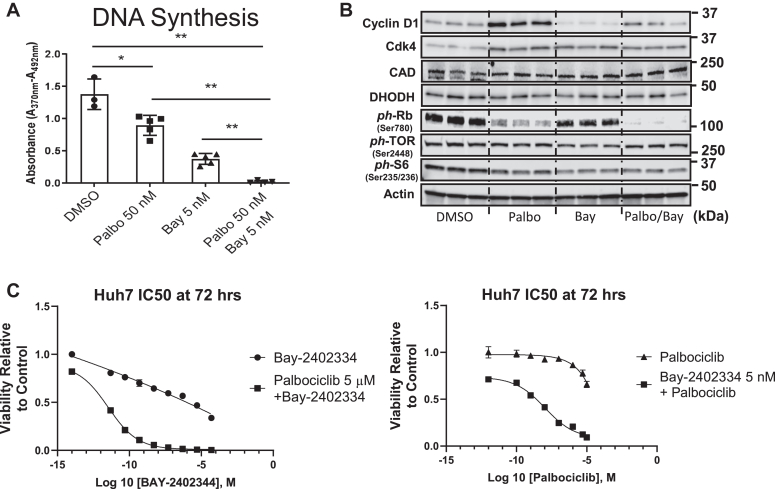
**Effect of Cdk4 and DHODH inhibition in HCC cells.** HuH7 cells were cultured in the presence of vehicle, palbociclib, and BAY2402234 as indicated. *A*, DNA synthesis as determined by BrdU uptake in cells treated with palbociclib (50 nM) and/or BAY2402234 (5 nM). *B*, Western blot of HuH7 cells cultured as in panel *A*. *C*, viability of cells treated with BAY2402234 at the indicated concentrations with and without palbociclib (5 μM, *left*), or with palbociclib at the indicated concentrations with and without BAY2402234 (5 nM, *right*). Cdk4, cyclin-dependent kinase 4; DHODH, dihydroorotate dehydrogenase; HCC, hepatocellular carcinoma.

Because of our novel finding that cyclin D1 promotes pyrimidine synthesis, we wondered whether combined inhibition of its main kinase partner (Cdk4) along with the downstream pyrimidine synthesis enzyme dihydroorotate dehydrogenase (DHODH) (Fig. 6*B*) would synergistically block cancer cell growth. In the HCC cell line HuH7, we used Palbociclib and BAY2402234 to inhibit Cdk4/6 and DHODH (respectively). At 48 h after treatment, Palbociclib (50 nM) and BAY2402234 (5 nM) each partially inhibited cell cycle progression as measured by DNA synthesis, while the combined drug treatment completely prevented DNA synthesis (Fig. 7*A*). Western blot analysis showed that neither drug affected phosphorylation of proteins in the TORC1 signaling pathway or DHODH expression (Fig. 7*B*). At 72 h after treatment, a higher dose of palbociclib (5 μM) synergistically enhanced cell killing by BAY2402234 (at doses of 1 nM or less), and BAY2402234 (5 nM) enhanced the effect of palbociclib (Fig. 7*C*). These studies indicate that inhibition of cyclin D1/Cdk4 markedly sensitives cells to further inhibition of the pyrimidine synthesis pathway.

## Discussion

Although cyclin D1 has been extensively studied, there is little information about how it regulates biosynthetic pathways required for proliferation and growth. Our prior studies have demonstrated that it represses key transcription factors that promote homeostatic metabolism in hepatocytes, including HNF4α, PPARα, and ChREBP (12, 14, 18), which in principle would allow for diversion of metabolic resources into biosynthesis and other needs of cell growth. In this study, we present evidence that cyclin D1 has substantial and multifaceted stimulatory effects on anabolic metabolism in hepatocytes. Cyclin D1 plays a pivotal role in physiologic hepatocyte proliferation (12, 13, 14, 15, 16, 17, 18), but is best known as an oncogene that is one of the most commonly overexpressed proteins in human cancers (21, 22). The studies outlined here, which were derived from unbiased metabolomic and proteomic analyses, demonstrate that cyclin D1 promotes a surprisingly broad portfolio of biosynthetic pathways in hepatocytes, which may be highly relevant to its role in normal and malignant cell proliferation.

In addition to its canonical role in activating Cdk4 and cell cycle progression, cyclin D1 regulates numerous other processes through Cdk-dependent and Cdk-independent mechanisms, including transcription, differentiation, migration, and metabolic function (46, 47, 48). The effect of cyclin D1 appears to be context- and cell type-dependent. For example, in multiple myeloma cells, cyclin D1 promotes hexokinase 2 expression, a regulator of glycolysis in these cells (49). In contrast, hepatocytes do not express hexokinase 2, but utilize glucokinase for this step of glycolysis, and cyclin D1 knockdown or KO increases the expression and activity of glucokinase through a HNF4α-dependent mechanism (14). As noted above, in human T-cell acute lymphoblastic leukemia cells, the closely related cyclin D3 protein (in complex with Cdk6) phosphorylates and inhibits the activity of two key enzymes in the glycolytic pathway, PFK1 and pyruvate kinase M2, thereby inhibiting glycolysis and redirecting glucose metabolites into the PPP; importantly, cyclin D1/Cdk4 did not have this effect (36). The studies presented here indicate that cyclin D1 has markedly distinct effects—by promoting glycolysis (*via* aldolase and GAPDH) as well as PPP activity (*via* G6PD).

Our prior studies have shown that short-term transduction of cyclin D1 into hepatocytes rapidly induces hepatocyte proliferation and liver growth (15, 16, 17), and transgenic models of cyclin D1 overexpression have demonstrated growth in various organs (50, 51, 52, 53). Thus, cyclin D1 appears to be sufficient to trigger the biosynthesis required for growth and proliferation, but to our knowledge, previous studies have not examined its effects on anabolic pathways in detail. In the current study, we used two different “-omics” approaches to gain insight into the mechanisms by which cyclin D1 promotes anabolic metabolism in hepatocytes. Proteomics analysis of cyclin D1-binding proteins revealed that this cell cycle protein forms complexes with numerous metabolic enzymes, including those involved in glycolysis and pyrimidine metabolism. Stable isotope-resolved metabolomics revealed that cyclin D1 depletion inhibited key steps of glycolysis, the PPP, and both purine and pyrimidine metabolism. The combined results offer new insight into how this key cell cycle protein and oncogene promotes both proliferation and the requisite biosynthesis for growth.

Previous studies have shown that cyclin D1 represses key elements of hepatic glucose metabolism including gluconeogenesis and glycogen synthesis (14, 19, 20). In this work, we focused on how cyclin D1 regulates metabolic pathways that utilize glucose for biosynthesis. Using several methodologies, we present data that cyclin D1 knockdown substantially inhibits glycolysis at the level of aldolase and/or GAPDH, as evidenced by the pronounced block in ^13^C incorporation from the glucose tracer into 3-carbon glycolytic metabolites (but not their 6-carbon precursors). This is corroborated by decreased activity of both aldolase and GAPDH in enzyme assays in the setting of cyclin D1 depletion. Furthermore, cyclin D1 binds to each of these proteins, and the cyclin D1/Cdk4 complex phosphorylates both in *in vitro* kinase assays. We hypothesize that cyclin D1/Cdk4 kinase may activate these enzymes by phosphorylation, but further study is required to decipher the precise mechanism(s) involved and to delineate how this affects intersecting metabolic pathways such as serine/glycine synthesis, one-carbon metabolism, and the tricarboxylic acid cycle.

Glucose flux through the oxidative PPP is generally upregulated in proliferating and malignant cells, and activity of the initial and rate limiting enzyme G6PD is regulated through complex and incompletely characterized mechanisms (37, 38). Although cyclin D1 depletion did not significantly affect G6PD protein levels, both the metabolomic analysis and activity assays indicated decreased activity of this enzyme. These data suggest that cyclin D1 promotes oxidative PPP flux *via* a posttranslational or allosteric mechanism during hepatocyte proliferation, which stimulates the production of ribose (PRPP) for nucleotide synthesis and NADPH for antioxidation.

The studies to trace the metabolic fate of glucose carbons also revealed that cyclin D1 plays a significant role in *de novo* purine and pyrimidine synthesis, which are essential for the markedly increased synthesis of DNA, RNA, and related metabolites during proliferation (39, 40). Cyclin D1 depletion inhibited purine ring synthesis and diminished the expression of the ATF4 transcription factor and enzymes that play key role in this process, including Mthfd2, Psat1, and Ppat. Interestingly, a similar pattern is seen in response to TORC1 inhibition (41). We have previously shown that cyclin D1 promotes growth and proliferation downstream of TORC1 in hepatocytes (16, 54). Although further study is required, cyclin D1 may be a key effector of TORC1 in regard to purine synthesis and other metabolic adaptations described here.

The metabolomics analysis found that inhibition of cyclin D1 expression led to a marked decrease in pyrimidine abundance, with nearly complete inhibition of pyrimidine ring synthesis. Expression of CAD, and its TORC1-dependent phosphorylation at Ser1859, were unchanged by cyclin D1 siRNA or KO. However, production of N-carbamoyl-L-aspartate was almost completely inhibited, despite the availability of the substrate aspartate, indicating marked inhibition of CAD enzyme activity. Our studies suggest that cyclin D1/Cdk4 phosphorylates CAD at a distinct site, and we surmise that this is required for full activation of this enzyme. Further studies are required to identify putative cyclin D1-dependent phosphorylation sites in CAD and their effect on enzyme activity, pyrimidine synthesis, and cell growth. However, the current studies demonstrate a novel link between cyclin D1 and pyrimidine synthesis.

Enhanced *de novo* pyrimidine synthesis is required for cell proliferation in many cancers (39, 40, 55, 56). Furthermore, recent analyses indicate that there is a strong link between human HCC and increased expression of genes involved in pyrimidine synthesis, suggesting that this pathway warrants further investigation as a therapeutic target (57, 58, 59). We surmised that drug inhibition of both Cdk4 (and thus indirectly, CAD) along with the next enzyme in the pyrimidine synthesis pathway, DHODH, would lead to pyrimidine starvation and thus effectively target HCC cell growth. Indeed, combined Cdk4 and DHODH inhibition appeared to be synergistic—in the presence of palbociclib, HCC cells were exquisitely sensitive to BAY2402234 (at doses of 1 nM or less, Fig. 7, C). It is likely that this drug combination perturbs other key metabolic pathways, including the electron transport chain complex 3 (which requires DHODH activity (60)), and thus several mechanisms may be at play. The potential synergy of clinically available Cdk inhibitors and high-potency selective DHODH inhibitors warrants further investigation.

In summary, the data presented here indicate that the key cell cycle protein cyclin D1 broadly promotes biosynthetic networks, and therefore plays a central role in governing the metabolic rewiring required for growth and proliferation in hepatocytes. Our prior studies have shown that cyclin D1 represses PPARα activity, lipolysis, lipophagy, and fatty acid oxidation (12, 13, 14). Thus, cyclin D1 appears to regulate a metabolic switch that reduces fatty acid oxidation and increases glycolytic and anabolic metabolism, which are common characteristics of proliferating and malignant cells. The induction of numerous biosynthetic pathways likely underlies the ability of cyclin D1 to promote hepatocyte proliferation and liver growth in the absence of other mitogenic stimuli and may contribute to its role in cancer. These studies support the concept that metabolic control is a key function of cyclin D1.

## Experimental procedures

### Cell culture and liver tissue harvest

The mouse hepatocyte AML12 cell line, human liver cancer cells HuH7, HepG2, and Sk-Hep1, and breast cancer MCF7 cells were cultured in 10% fetal bovine serum and treated with control or cyclin D1, or transduced with ADV-D1 (or a control ADV) as previously described (12, 13, 18). Cell culture, BrdU incorporation, and viability assays were performed as previously described (12, 13, 18, 61). HuH7 cells were treated with palbociclib and BAY2402234 (MedChemExpress) at the concentrations shown.

Animal studies were approved by the Institutional Animal Care and Use Committee at the Minneapolis VA Health Care System. Liver tissue was harvested at rest (0 h) or 42 h after two-thirds PH from cyclin D1^fl/fl^ mice dosed 1 week prior with adeno-associated virus serotype 8 (AAV8)-thyroxine-binding globulin (TBG)-enhanced GFP (AAV8-TBG-GFP, the control vector) or with AAV8-TBG-CRE (encoding the Cre recombinase) to achieve acute hepatocyte-specific KO as previously described (14).

### Protein isolation, Western blot, and reverse phase protein array

Protein isolation from cells and tissue and Western blot were done as previously described (13, 14). Additional antibodies for Western blot included the following: CAD (93925), ph-CAD (67235), G6PD (12263), GAPDH (5174), ph-RB (35908), ph-S6 (2211), ph-S6K1 (9205), and ph-mTOR (5536) from Cell Signaling Technology; Aldolase B (18065-1-AP<ATF4 (10835-1-AP), and PSAT1 (10501-1-AP) from ProteinTech. Precast Phos-Tag gels for Western blot were obtained from Wako and used as recommended by the manufacturer. Western blot images and quantification were performed using the Licor Odyssey XF Imaging System. Quantified protein expression was normalized to actin expression in each specimen.

For reverse phase protein array, protein extracts (at 0.2–0.5 mg/ml) from the cell culture experiments were printed as two drops per spot onto a slide coated with 16 nitrocellulose membrane pads (Grace Bio-Labs) using a microarray printer (Arrayjet). As described previously (62), slides were incubated in Fast Green protein stain and scanned at 670 nm emission wavelength with InnoScan 710 AL microarray scanner (Innopsys, Inc) to determine the amount of protein deposited per sample spot. Slides were then incubated in the blocking buffer (5% fetal bovine serum in Tris-buffered saline with 0.1% Tween 20 detergent), followed by incubation in a primary antibody diluted 1:100 in the blocking buffer (see below for vendor and dilution information) against a target protein for 2 h at 20 °C, washing in TBST, incubation with fluorescent secondary antibody (LICOR-IRDye 800) at 1:1000 dilution in the blocking buffer for 1 h at 20 °C, washing in TBST, and drying *via* vacuum suction. Slides were scanned at 700 nm emission wavelength with InnoScan 710 AL. Fluorescence image analysis of spots was done using the Innopsys Mapix software. Background fluorescence for each spot was subtracted from the fluorescence signal for that spot followed by normalization to the Fast Green signal. Normalized signals were averaged across replicates (n = 6). The following antibodies were used: PSAT1 (Proteintech 10501-1-AP), PPAT (Proteintech 15401-1-AP), MTHFD2 (Proteintech 12270-1-AP), PFKL (Invitrogen PA5-21685), PFKM (Proteintech 55028-1-AP), and PFKP (Proteintech 13389-1-AP).

### ECAR and OCR measurements

The Seahorse XFe96 analyzer (Agilent) was used to measure ECAR and OCR according to the manufacturer’s instructions. Both XF Mito Stress Test and XF Glycolytic Rate assays were performed to investigate mitochondrial and glycolytic function. In brief, AML12 cells were seeded with 15,000 per well overnight in a 96-well XF cell culture microplate in growth medium. After 48 h of siRNA transfection or ADV transduction, cell culture medium was replaced 1 h before the assay with XF base medium supplemented with 1 mmol/L sodium pyruvate, 10 mmol/L glucose, and 2 mmol/L glutamine. For XF Mito Stress Test, OCR was analyzed at basal conditions and after sequential injections of oligomycin (1 μmol/L), carbonyl cyanide-4-(trifluoromethoxy)phenylhydrazone (FCCP) (2 μmol/L), and antimycin/rotenone (0.5 μmol/L). For glycolytic rate assay, the proton efflux rate was measured at basal conditions and following rotenone and antimycin A (complex I and II inhibitors) and 2-DG (glycolysis inhibitor). Glycolytic Rate Assay Report Generator was utilized to calculate proton efflux rate. Results were normalized to cell number in each well.

### Biochemical assays

Cells were grown on 12-well plates. The media were replaced with fresh media at 24 h after siRNA treatment. After 48 h, the media were harvested for glucose and lactate assays. Media glucose levels were determined using a glucose assay kit (CBA086) and lactate levels measured using a lactate assay kit (MAK064) obtained from Sigma-Aldrich. Results were normalized to the cellular protein levels in each well.

Assay kits for aldolase (ab196994, Abcam) and GAPDH (MAK277, Sigma-Aldrich) were used as instructed by the manufacturers.

### Statistical analysis

Replicates shown in the figures represent independent experiments done in parallel. Data are expressed as mean ± SD. Statistical analysis was performed using GraphPad software (GraphPad Software, Inc; www.graphpad.com). Comparisons between two groups were made by Student *t* test, and significant differences were noted (∗*p* < 0.05; ∗∗*p* < 0.01; and ∗∗∗*p* < 0.001).

### Proteomic analysis of cyclin D1-binding proteins

Mice were injected with an ADV encoding cyclin D1 with C-terminal FLAG and HA tags that was produced by Vector Biolabs or ADV-cyclin D1 (with no tags, used a negative control for affinity purification) and harvested after 24 h as previously described (15, 16, 17). Protein extracts from the liver were immunopurified using Anti-FLAG affinity beads followed by elution using FLAG peptide as described by the manufacturer (F3290, Sigma-Aldrich). These eluates were then purified using Anti-HA affinity beads with elution according to the manufacturer’s instructions (A2095, Sigma-Aldrich).

The purified proteins were further processed at the University of Minnesota’s Center for Mass Spectrometry and Proteomics. For each immunoprecipitated sample a 32 μl aliquot of sample was mixed with 12.5 μl 4X Laemmli SDS loading buffer. The samples were heated at 95 °C for 5 min and loaded into separate wells on a Bio-Rad 10% Criterion TM Tris–HCl gel. The samples were run at 25 mA constant current for 30 min. The gel was stopped, fixed with 40% ethanol, and 10% acetic acid for 30 min. The gel was washed with LC-MS grade water for 5 min, repeated twice. The gel was then stained with Thermo Fisher Scientific’s Imperial Protein Stain for 1 h and destained with LC-MS grade water overnight. Equal gel area regions for each sample were excised and subjected to in-gel proteolytic digestion as described previously (63) with the following difference. During the alkylation step, 55 mM iodoacetamide was used instead of 55 mM methyl methanethiosulfonate. Post digestion, each sample was then cleaned with a MCX STAGE tip (64). Eluates were vacuum dried. The cleaned samples were run on the Thermo Orbitrap Velos.

We resuspended the dried peptide pellets in load solvent (97.99:2:0.01, water:acetonitrile:formic acid) and analyzed approximately 400 ng of material on the LTQ Orbitrap Velos using HCD [higher energy collision induced dissociation] activation and FT [Fourier transform] detection (Thermo Fisher Scientific) system with an Eksigent 1D NanoLC as previously described (65) with the following revisions: the capillary liquid chromatograph column inner diameter was 100 μm, the liquid chromatograph gradient was 2 to 8% B solvent from 0 to 2 min and 5 to 35% B from 2 to 67 min, 35 to 90% B at 68 min, then held at 90% B until 85 min with a flowrate of 330 nl/min; lock mass was not invoked; MS1 survey scan was 380 to 1800 m/z; MS1 maximum injection time was 300 msec, dynamic exclusion list size was 200, duration was 30 s, and the tolerance was ±15 ppm.

We analyzed the tandem mass spectrometry (MS/MS) data using Sequest (66) (Thermo Fisher Scientific in Proteome Discoverer 2.2.0.388, https://www.proteomesoftware.com). Sequest was set up to search the Mus (taxon ID 10088) Reference Sequence protein database downloaded from NCBI on April 1, 2015, after concatenation of the common lab contaminants protein sequences from https://www.thegpm.org/crap/. The total number of protein sequences was 57,928. The search parameters included: trypsin enzyme with full specificity; fragment ion mass tolerance of 0.1 Da; precursor ion tolerance 50 ppm; carbamidomethyl cysteine as a fixed amino acid modification; acetylation of protein N terminus, oxidation or dioxidation of methionine, pyroglutamic acid modification of glutamine, and asparagine deamidation as variable modifications.

Scaffold (version 4.9, Proteome Software Inc, https://www.proteomesoftware.com/products/scaffold-5) was used to validate MS/MS based peptide and protein identifications. Peptide identifications were accepted if they could be established at greater than 81.0% probability to achieve a false discovery rate (FDR) less than 1.0% by the Scaffold Local FDR algorithm. Protein identifications were accepted if they could be established at greater than 15.0% probability to achieve an FDR less than 1.0% and contained at least two identified peptides. Protein probabilities were assigned by the Protein Prophet algorithm (67). Proteins that contained similar peptides and could not be differentiated based on MS/MS analysis alone were grouped to satisfy the principles of parsimony. Proteins sharing significant peptide evidence were grouped into clusters.

### ^13^C-glucose labeling and metabolite analysis in AML12 cells

Glucose- and glutamine-free Hams/F12 media was obtained from Gibco Thermo Fisher Scientific. The medium was supplemented with 2.5 mM glutamine and 17.5 mM [U-^13^C]-glucose (Cambridge Isotope Laboratories) for the final 24 h. At the end of the incubation, cells were washed with ice-cold PBS three times followed by extraction with acetonitrile/water/chloroform (V/V 2:1.5:1) and separated into polar, lipid, and protein fractions as described previously (68, 69). Three replicate separate samples were analyzed for both the cyclin D1 knockdown and control siRNA groups.

For ion chromatography coupled with IC-UHR FT MS, methanol (HPLC grade, ≥99.9%, Sigma-Aldrich) was used as the make-up solvent providing after ion chromatography to assist electrospray in the mass spectrometer. Nano pure water was obtained from a Milli-Q Integral Water Purification System (Thermo Fisher Scientific). Ion chromatography was carried out using a Dionex ICS5000+ system equipped with a dual pump, an eluent generator, an autosampler and a detector/chromatography module. An IonPac AG11-HC-4 μm guard column (2 × 50 mm) followed by an IonPac AS11-HC-4 μm RFIC&HPIC (2 × 250 mm) analytical column was used with a constant temperature at 35 °C and the column flow rate was kept at 0.38 ml/min. The eluent was injected into a Thermo Fusion Orbitrap mass spectrometer using full scan negative ion mode with a nominal resolution of 360,000 at m/z = 200 as previously described (70). Metabolites were identified and quantified using TraceFinder version 3.3 (Thermo Fisher Scientific; www.thermofisher.com) software using a mix of 88 standards and corrected for natural abundance ^13^C contributions (71).

NMR spectra of polar metabolites were recorded at 14.1 T on an Agilent DD2 spectrometer with automation in 1.7-mm tubes in a 3-mm inverse ^1^H^13^C^15^N triple resonance cold probe at 15 °C with an acquisition time of 2 s and a presaturation delay of 4 s for ^1^H experiments (PRESAT), using a weak transmitter rf field for saturating the strong solvent signal. 1D ^1^H-heteronuclear single quantum coherence spectra were recorded with an acquisition time of 0.2 s with adiabatic decoupling and a relaxation delay of 1.8 s. Samples were maintained at 6 °C prior to NMR analysis. Compounds were identified from chemical shifts and splitting patterns as previously described using our in-house database (69) and those of Human Metabolome Database (72). We used a targeted analysis for a small number of abundant compounds as per protocol for non-stable isotope-resolved metabolomics experiments. Whenever possible, we also estimated the 13C enrichment at individual positions such as lactate and Ala methyl groups and glucose H1. Data were analyzed using MNova v 12.0 (Mestrelab Research; mestrelab.com). Free induction decays were zero filled to 256k points (PRESAT) or 16k points (heteronuclear single quantum coherence) and apodized using a cosine-squared function with a 1 Hz (PRESAT) or 4 Hz line broadening (heteronuclear single quantum coherence) exponential function prior to Fourier transformation. The spectra were phased and baseline corrected with a simple third order polynomial. Concentrations were determined by peak integration using the curve fitting routine of MNOVA with normalization to the 2,2′-dimethylsilapentane-5-sulfonate resonance at 0 ppm, with corrections for partial saturation as described (69). Concentrations are presented as mean ± sem. Isotopomer distributions were determined as previously described (17).

### Cyclin D1/Cdk4 kinase assays

For nonradioactive *in vitro* kinase assays, 100 ng of recombinant cyclin D1/Cdk4 (ab55695, Abcam) was combined with 1 μg of recombinant Aldolase B (ab123165, Abcam) or GAPDH (ab82633, Abcam) along with 200 μM ATPγS (ab138911, Abcam) in a buffer of 50 mM Hepes, 10 mM MgCl_2_, 1 mM DTT, 1 mM EGTA, and 0.1 mM NaF for 30 min at 30 °C. Alternatively, FLAG-HA-CAD isolated from the mouse liver (as below) was used as substrate. The kinase reaction mixture was alkylated by adding p-Nitrobenzyl mesylate (ab138910, Abcam, final concentration 2.5 nM) for 1 h at room temperature. This was run for Western blot as above, and using anti-thiophosphate ester antibody (ab92570, Abcam).

Using a previously described FLAG-HA-CAD plasmid construct (43) (obtained from Addgene), an adenoviral vector was created by Vector Biolabs. This was used to transduce hepatocytes in mice as previously described (15) 2 days prior to harvest. Mice were fasted overnight before harvesting the liver, and FLAG-purification of lysates was performed as above. The isolated FLAG-HA-CAD protein was then used as substrate for kinase assays above.

## Data availability

All data are included in the manuscript.

## Supporting information

This article contains supporting information.

## Conflict of interest

The authors declare that they have no conflicts of interest with the contents of this article.

## Supporting information

Dataset

Supplemental Fig. S1 **Cyclin D1 depletion inhibits glycolysis in human cancer cell lines.** The indicated cell lines were cultured in serum and treated with cyclin D1 or control siRNA. *A*, Western blot of cyclin D1. *B*, DNA synthesis as determined by BrdU uptake, glucose uptake from the media, and lactate production as measured in the media.

Supplemental Fig. S2 **AML12 cells were culture as in**Figure 3**.***A*, expression of Pfk isoforms as measured by reverse phase protein array analysis. *B*, ATP/ADP and ATP/AMP ratios based on concentrations assess by MS from cells that were not labelled with ^13^C-glucose. In this figure, only PFKL protein levels were significantly different in the two groups.
