# Supplementary figures and images for: Cyclin D1 extensively reprograms metabolism to support biosynthetic pathways in hepatocytes
Source: J Biol Chem. 2023 Oct 28;299(12):105407. doi: 10.1016/j.jbc.2023.105407 (PMC10687208; doi:10.1016/j.jbc.2023.105407)

# Sup. Fig 1

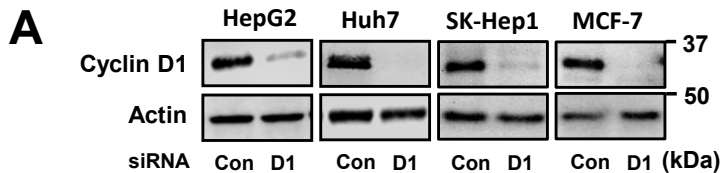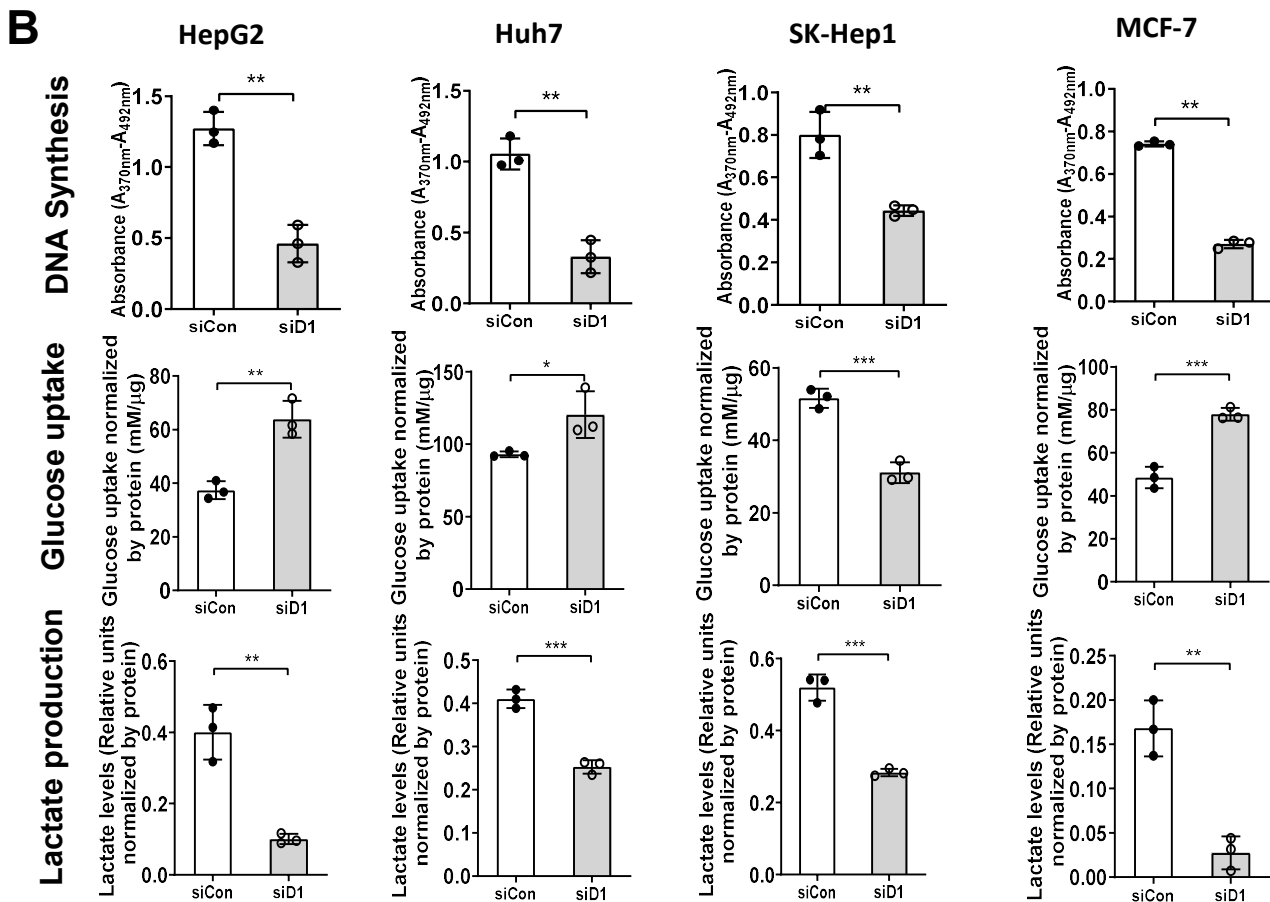

Supplement: Supplemental Fig. S1 — Cyclin D1 depletion inhibits glycolysis in human cancer cell lines. The indicated cell lines were cultured in serum and treated with cyclin D1 or control siRNA. A, Western blot of cyclin D1. B, DNA synthesis as determined by BrdU uptake, glucose uptake from the media, and lactate production as measured in the media. [file mmc2.pdf]

# Sup. Fig 2

## A

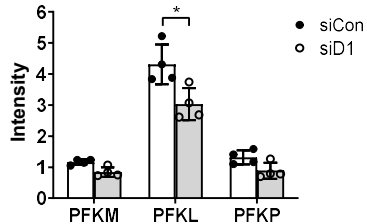

## B

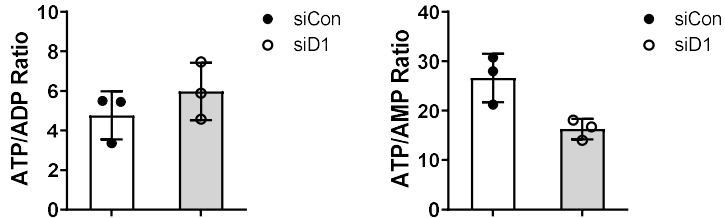

Supplement: Supplemental Fig. S2 — AML12 cells were culture as inFigure 3.A, expression of Pfk isoforms as measured by reverse phase protein array analysis. B, ATP/ADP and ATP/AMP ratios based on concentrations assess by MS from cells that were not labelled with 13C-glucose. In this figure, only PFKL protein levels were significantly different in the two groups. [file mmc3.pdf]
